# Annotations

**Published:** 1897-11-13

**Authors:** 


### Annotations.

### Massage by the Blind.

The suggestion has been made, and with a certain show of reason, that the practice of massage might well be undertaken by the blind. The extreme delicacy of touch which, as is well known, blind people often possess, would undoubtedly give them a considerable advantage in this sort of work so far as mere manipulation is concerned; and it seems not improbable that some sensitive people might be willing to submit to massage by one who could not see, while they would hesitate to expose themselves and their little deficiencies to critical eyes. Of this, however, we do not feel very confident, and what we would point out is that massage, at its best, is not a mere local manipulation, and that although in certain cases, as of muscular atrophy or writers' cramp, a blind masseur might be very usefully employed, the case is quite different when general massage is in question. Then it becomes necessary to take cognizance of many general signs, so as to determine the effect which the process is having upon the patient, and for these purposes observation by the eye is absolutely essential. The masseur or the masseuse should be able to judge how things are going on without asking questions and without directing attention to any symptom of fatigue, and for this purpose every sense must be alert. We think, therefore, that massage by the blind will have but a limited utility, although in its own field it may possess certain advantages.

### The Increase of Suicide.

It is by no means comforting to find that, although the deaths which take place from fevers, from tuberculosis, and suchlike scourges are diminishing, those from suicide and diseases of the nervous system are increasing. It is difficult indeed, to withhold the thought that there may be some connexion between that increase in the power of the healing art and that extension of charitable efforts to save and to prolong the lives of the feeble and the sick, which are such marked characteristics of modern times, and the apparent increase in that particular class of diseases which are most distinctly hereditary, and especially of that final nervous defect which leads a man to deprive himself of life. The Harveian Orator for this year has shown how civilisation in the modern sense of the term is founded upon science, and therefore will endure. But when science devotes itself to saving life, while to the individual it does good, it is by no means certain that it does good also to the race; and when the saving of life means the saving from their natural fate of those who are unfit, unaided, to attain maturity, to support themselves, or to resist the rougher influences which the race must resist if it is to continue, then we may question how far science is doing good to the community. An American writer, Dr. Lawrence Irwell, says: "Never in the history of the world was there a time when such strenuous efforts were made to prolong the lives of the absolutely unfit, that they might have an opportunity of reproducing their kind. Never was there a race which suffered as the English-speaking race is now suffering from the fertility of the worst specimens of humanity. With each generation the vitality of the community is being reduced by its manner of life, and in order to enable it to continue the fight against the inevitable laws of nature all sorts of artificial aids have been invented. False teeth, spectacles, ear trumpets, wigs—to say nothing of predigested foods—are a few of the contrivances with which we are trying to carry out the pernicious doctrine of the survival of the unfittest." Among other signs of the degeneracy which is said to follow in the steps of all our endeavours to help the sick and the afflicted—who, according to the doctrine of heredity, we ought to leave to perish—is the growing number of suicides, of people who if not, as the jury generally has it, "temporarily insane," are at least of such a degenerate fibre that they cannot stand up against the worries of the world.

### The Tooth Brush.

It is but a little thing, yet on its proper use depends much of the happiness of modern man. Why civilised teeth should be so rotten is a question which has often been debated, and probably the true answer is more complex than some would think. Many good mothers are content to put all toothache down to lollypops; but that sugar in itself is not responsible for bad teeth is proved by the splendid "ivories" often possessed by negroes who practically live upon the sugar-cane, and thrive upon it too, during the whole of the season when it is in maturity. Dental decay is common enough, however, among negroes in towns, and it seems clear that the caries of the teeth, which is so common among most civilised races, is due not to any particular article of diet so much as to digestive and nutritive changes imposed upon us by our mode of life, and to some extent by the fact that by hook or by crook we do somehow manage to live, notwithstanding our bad teeth; whereas, in a state of nature, the toothless man soon dies. Recognising, then, that until the time arrives when some great social reformer either mends or ends our present social conditions our teeth will tend to rot, and that, whatever the predisposing causes, the final act in the production of caries is the lodgment of microbes on and around the teeth, we see that for long to come the tooth brush will be a necessity if the health is to be maintained. It is only by the frequent use of this little instrument that those minute accumulations can be removed which are at the root of so much mischief. A few elementary lessons in bacteriology would, we fancy, greatly startle many people, and certainly would show them the futility of trusting to one scrub a day. The fact is, that if people, instead of looking at the tooth brush from an æsthetic point of view, and scrubbing away with tooth powders (!) to make their front teeth white, would regard it merely as an aid to cleanliness, they would see that the time to use it is after meals and at night, not just in the morning, only, when the *débris* left from the day before has been fermenting and brewing acid all night through. They would also see how inefficient an instrument the common tooth brush is unless it is used with considerable judgment. One of the secondary advantages of spending a good deal of money on dentistry is that at least one learns the value of one's teeth. By the time we have got them dotted over with gold stoppings and gold crowns we learn to take care of them, even although that may involve the trouble of cleaning them more than once a day and using perhaps more than one brush for the purpose.

